# Impact of Environmental Factors on *Legionella* Populations in Drinking Water

**DOI:** 10.3390/pathogens4020269

**Published:** 2015-05-19

**Authors:** David Otto Schwake, Absar Alum, Morteza Abbaszadegan

**Affiliations:** 1 School of Life Sciences, Arizona State University, Tempe, AZ 85281, USA; E-Mail: david.schwake@asu.edu; 2 School of Sustainable Engineering and the Built Environment, Arizona State University, Tempe, AZ 85281, USA; E-Mail: alum@asu.edu

**Keywords:** *Legionella pneumophila*, survival, drinking water, distribution system, temperature, biofilm

## Abstract

To examine the impact of environmental factors on *Legionella* in drinking water distribution systems, the growth and survival of *Legionella* under various conditions was studied. When incubated in tap water at 4 °C, 25 °C, and 32 °C, *L. pneumophila* survival trends varied amongst the temperatures, with the stable populations maintained for months at 25 °C and 32 °C demonstrating that survival is possible at these temperatures for extended periods in oligotrophic conditions. After inoculating coupons of PVC, copper, brass, and cast iron, *L. pneumophila* colonized biofilms formed on each within days to a similar extent, with the exception of cast iron, which contained 1-log less *Legionella* after 90 days. *L. pneumophila* spiked in a model drinking water distribution system colonized the system within days. Chlorination of the system had a greater effect on biofilm-associated *Legionella* concentrations, with populations returning to pre-chlorination levels within six weeks. Biofilms sampled from drinking water meters collected from two areas within central Arizona were analyzed via PCR for the presence of *Legionella*. Occurrence in only one area indicates that environmental differences in water distribution systems may have an impact on the survival of *Legionella*. These results document the impact of different environmental conditions on the survival of *Legionella* in water.

## 1. Introduction

The *Legionella* genus contains over 50 species of gammaproteobacteria [1], many of which are capable of producing respiratory illness in humans. These bacteria are ubiquitous in both natural and artificial water systems, where their growth is often associated with biofilms. While not necessary for growth, biofilm habitation provides *Legionella* protection from harmful substances such as disinfectants as well as more ready access to eukaryotic host organisms thought to be required for their replication in the environment [2]. The typical hosts for these bacteria are thought to be protozoan biofilm grazers [3], notably amoeba such as *Hartmanella* [4]. Despite this, *Legionella* are also capable of infecting a wide variety of eukaryotes, with humans being a potential incidental host. Although able to survive in a wide range of temperatures, *Legionella* typically require relatively warm environments—often found in anthropogenic water systems—to reach population levels capable of posing public health risks to humans [5].

The first reported outbreak of legionellosis occurred at an American Legion convention in Philadelphia, PA during the summer of 1976. Several months later, the causative agent of the pneumonia cases linked to the outbreak was found to be a previously unidentified environmental bacterium, *Legionella pneumophila* [6]. While various forms of legionellosis infections have been documented, the overwhelmingly vast majority result in one of two respiratory diseases: Pontiac fever is a self-limiting febrile disease that is poorly documented, whereas Legionnaires’ disease is a potentially deadly pneumonia and is often reported [7]. Though a majority of confirmed Legionnaires’ disease cases occur in the hospital setting, both community-acquired and nosocomial outbreaks of legionellosis occur regularly [8]. Drastically increasing rates of legionellosis outbreaks have led to *Legionella* being responsible for more drinking water related disease outbreaks in the United States than any other microbe [9], highlighting the importance of the development and implementation of measures to prevent this disease.

As *Legionella* require temperatures above 20 °C to grow, contamination is often associated with heated artificial water systems such as cooling towers, spas, and hot tap water lines [10]. As a result, the vast majority of legionellosis prevention measures are aimed at treatment of these high-temperature systems. Although the warm temperatures necessary for *Legionella* replication are more likely to occur in these environments, non-heated engineered water systems are also common sources of outbreaks [11]. Due to this fact, the impact of drinking water main distribution systems on the transmission of legionellosis warrants examination. While a large number of studies have been aimed at investigating the incidence and survival of *Legionella* at the in-premise level, a relatively small amount of research has been focused on *Legionella* within main distribution systems [12], leading to a knowledge gap about their interactions with these systems. The objectives of this study were to elucidate the ecology of *Legionella* in drinking water distribution systems via the following experiments: (1) a bench-scale experiment to measure the capability for *Legionella* to survive in tap water at varying temperatures; (2) a bench-scale experiment to determine the association and survival of *Legionella* within biofilms on various water pipe materials; (3) a pilot-scale experiment examining the interactions between *Legionella* in flowing water and biofilms of a model drinking water distribution system (MDS); and (4) a field study of the occurrence of *Legionella* in drinking water distribution system biofilms from residential water meters.

## 2. Results

### 2.1. Survival of *Legionella* in Tap Water at 4 °C, 25 °C, and 32 °C

**Figure 1:**
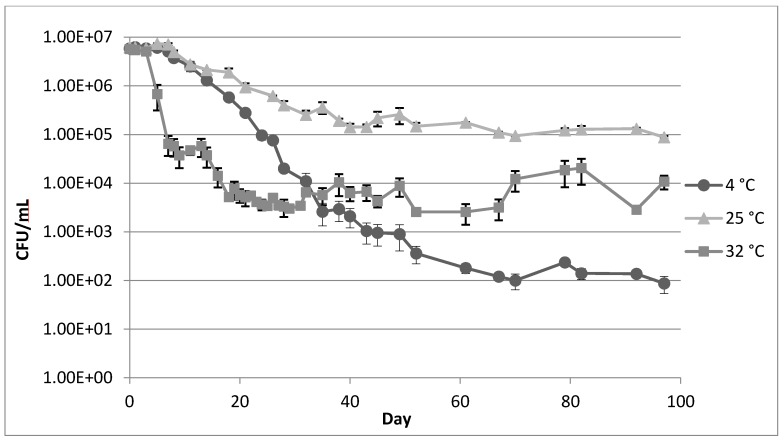
*L. pneumophila* concentration in 50 mL tap water incubated at 4 °C, 25 °C, and 32 °C over 97 days after inoculation with 3 × 10^7^ cells. Day 0 represents sampling performed 2 h post inoculation. Error bars indicate standard error between two replicate cultures.

During the first week of incubation, no significant decrease in the concentration of *Legionella* was observed at any temperature (Figure 1). At 4 °C, after the first week of a steady state, *Legionella* concentration decreased at an exponential rate for two months before stabilizing at 10^2^ colony forming units (CFU)/mL for the remainder of the study period. At 25 °C, *Legionella* concentration decreased exponentially for 30 days and stabilized at approximately 10^5^ CFU/mL. At 32 °C, *Legionella* concentration decreased at an exponential rate for 18 days and relatively stabilized at approximately 5 × 10^3^ CFU/mL. After the initial decrease, the variation in concentration was more pronounced at 32 °C than for the other temperatures. After 11 months, *Legionella* concentrations at 4 °C, 25 °C, and 32 °C were <1 CFU/mL, <1 CFU/mL, and >10^3^ CFU/mL, respectively, suggesting that long-term survival of *Legionella* was supported by the higher temperature.

### 2.2. Association of *Legionella* within Biofilms on a Variety of Pipe Coupons

**Figure 2:**
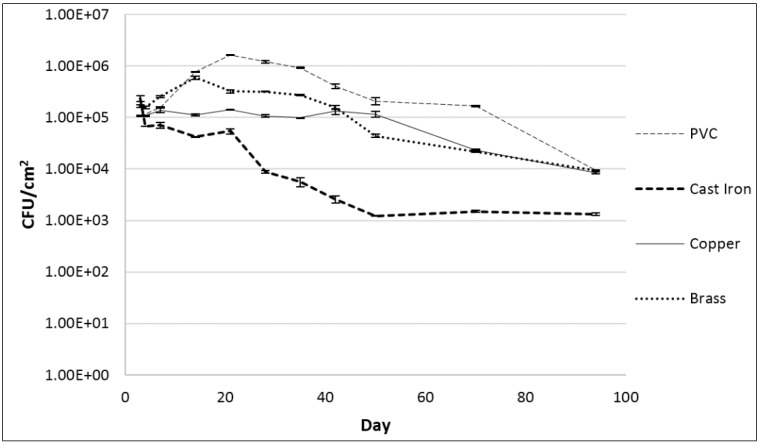
*L. pneumophila* concentrations in biofilms on coupons submerged in tap water incubated at 25 °C. Error bars indicate standard error between duplicate samples of each culture.

**Figure 3:**
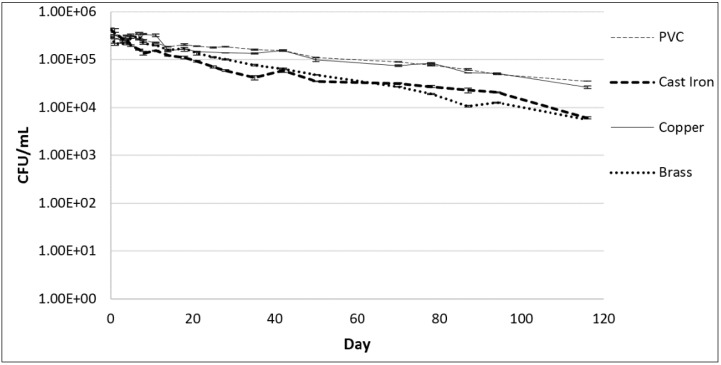
*L. pneumophila* concentrations in tap water containing the coupons incubated at 25 °C. Error bars indicate standard error between duplicate samples of each culture.

For all pipe materials, similar *Legionella* biofilm association (10^5^ to 2.7 × 10^5^ CFU/cm^2^) was observed three days after inoculation (Figure 2). After the initial three days, concentrations in biofilms formed on cast iron decreased before stabilizing at 1.2 × 10^3^ CFU/cm^2^ for the duration of the study, resulting in a 2-log reduction. Concentrations on copper remained stable near 10^5^ CFU/cm^2^ until day 50 and then decreased until day 90. Concentrations on brass increased seven-fold to a maximum of 7.6 × 10^5^ CFU/cm^2^ by day 14, and then steadily decreased until day 90. Concentrations on polyvinyl chloride (PVC) followed a similar trend to brass, increasing to 1.6 × 10^5^ CFU/cm^2^ by day 21 before decreasing. Final concentrations on copper, brass, and PVC were similar at day 90, each near 10^4^ CFU/cm^2^, approximately a 1-log reduction from the initial spiked concentrations. *Legionella* levels in water surrounding each coupon (Figure 3) eventually decreased, with final concentrations of 1.3 × 10^3^, 2.1 × 10^3^, 4.9 × 10^3^, and 4.9 × 10^3^ CFU/mL for brass, cast iron, copper, and PVC, respectively. Concentrations were stable for 3, 8, 8, and 14 days in water surrounding the cast iron, copper, brass, and PVC coupons, respectively.

### 2.3. Growth and Survival of *Legionella* in a Model Drinking Water Distribution System 

**Figure 4:**
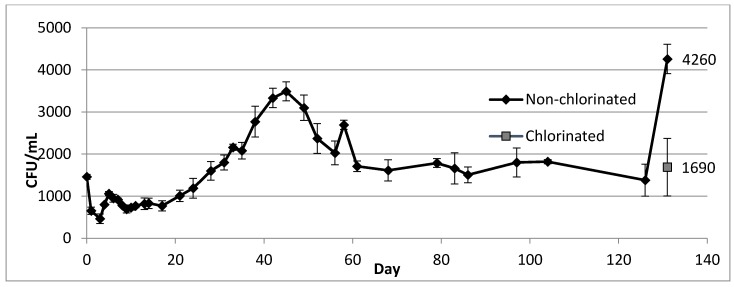
*L. pneumophila* in flowing water of a model drinking water distribution system over a period of 131 days after inoculation with 10^7^ cells. **Note:** Day 0 corresponds to sampling performed 2 h after inoculation. The gray square at day 131 corresponds to sampling performed 2 h after chlorination. Error bars indicate standard error of samples collected from two separate ports before and after flushing with 1 L of water.

**Figure 5:**
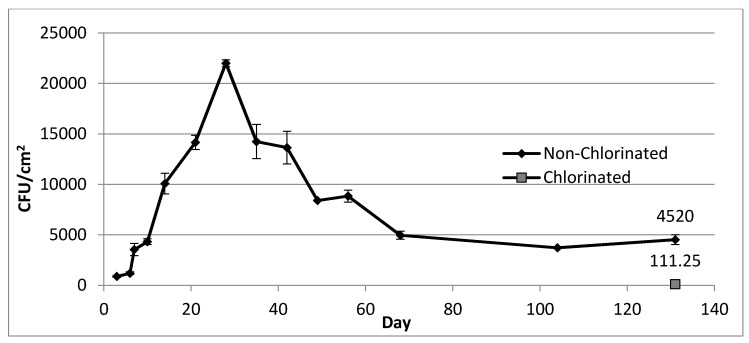
*L. pneumophila* in biofilms of a model drinking water distribution system over a period of 131 days after inoculation with 10^7^ cells. **Note:** The first data point corresponds to sampling performed three days post inoculation. The gray square at day 131 corresponds to sampling performed 2 h after chlorination. Error bars indicate standard error from two biofilm samples.

Within hours of inoculation, similar *Legionella* concentrations (approximately 1.5 × 10^3^ CFU mL^−1^) were detected in samples collected from both sampling ports (Figure 4) of the MDS. During the first week, *Legionella* concentration rapidly fluctuated and then stabilized at approximately 8 × 10^2^ CFU mL^−^^1^. From day 20 through day 45, a steady increase in concentration was observed, with a peak concentration of 3.5 × 10^3^ CFU mL^−1^, followed by a steady decrease until day 60. After this point, *Legionella* concentration stabilized at approximately 1.6 × 10^3^ CFU mL^−1^ until day 126. At day 131 a concentration of 4.3 × 10^3^ CFU mL^−1^ was recorded, followed by 1.7 × 10^3^ CFU mL^−1^ after chlorination. Three days after inoculation of the MDS, an initial biofilm concentration of 8.8 × 10^2^ CFU cm^−2^ was measured (Figure 5). Concentration within biofilm samples increased steadily, with a peak at 2.2 × 10^4^ CFU cm^−2^ on day 28, followed by a steady decrease until reaching a stable concentration near 4.4 × 10^3^ CFU cm^−2^ on day 68 until day 131. After chlorination, biofilm concentrations (averaged from coupons and pipe loop segments) decreased to 1.1 × 10^2^ CFU mL^−1^. After six weeks, concentrations in both biofilms and the MDS flowing water increased to pre-chlorination levels. In addition, culturable *Legionella* were detected for over a year in the MDS, with approximately 1.5 × 10^3^ CFU mL^−1^ in flowing water and 2 × 10^3^ CFU cm^−2^ in biofilm samples 13 months after the initial inoculation.

### 2.4. Presence of *Legionella* in Residential Water Meter Biofilms 

Molecular analysis of the biofilms samples tested from the 35 water meters collected from Area A confirmed the presence of *Legionella* in 9 m, with five samples also testing positive for *L. pneumophila* (Table 1). None of the 32 m collected from area B tested positive. All attempts at culture confirmation for viable *Legionella* were unsuccessful due to high levels of non-*Legionella* bacterial growth.

**Table 1:** *Legionella* and *L. pneumophila* occurrence via PCR in residential water meter biofilms.

Sample Site	*Legionella* spp. Positive	*L. pneumophila* Positive
System A	26% (9/35)	14% (5/35)
System B	0% (0/32)	0% (0/32)

## 3. Discussion 

### 3.1. Survival of *Legionella* in Tap Water at 4 °C, 25 °C, and 32 °C

Data from the initial three months of the experiment indicate a more stable population of *Legionella* at 25 °C compared to 4 °C and 32 °C. Conversely, culturable cells were only detected at 32 °C after 11 months. While periods of population maintenance were eventually established at each temperature, the cultures incubated at 25 °C reached a steady state quicker and with higher concentrations than at 4 °C or 32 °C. *Legionella* replication has been demonstrated to be faster with increasing temperature [13], raising the question as to why higher concentrations were observed in the initial three months of the experiment at 25 °C. The increased survival of the *Legionella* population observed at a lower temperature (25 °C *versus* 32 °C) during the initial three months of incubation may have been due to lowered metabolically linked cell turnover or similar factors. Cell cycling may have also contributed to the differences in concentrations observed at the two temperatures, as the highly pleomorphic *Legionella* spp. are known to alter cellular morphology in response to environmental stresses to aid survival under extreme conditions [14]. High temperatures are known to induce formation of resilient (yet often non-culturable) filamentous *Legionella* [15], which may have factored into the increased long-term survival observed at higher temperature. The rapid decrease in concentrations observed during the initial days of the experiment may have been linked to this phenomenon of the formation of viable but non-culturable *Legionella*. This is particularly relevant in regards to filamentous cells, as a single multinucleate filament capable of giving rise to dozens of individual bacilli under specific environmental conditions may only produce a single colony when cultured. Regardless of cell cycling dynamics, replication could have contributed to the increased survival observed at both higher temperatures compared to 4 °C. This appears to have been demonstrated by multiple concentration increase in *Legionella* population in the 32 °C incubation, including a one log increase between days 67 and 82. No such increase in population was recorded for the cultures incubated at the other temperatures. It should be noted that the long-term survival and population stabilization from this experiment occurred in the absence of mature biofilm formation, as the frequent agitation of the tubes prior to sampling should have disrupted and minimized biofilm formation.

### 3.2. Association of *Legionella* within Biofilms on a Variety of Pipe Coupons 

The association of *Legionella* within biofilms on each coupon examined demonstrates the capability of this organism to establish a population on a wide variety of pipe materials. Two materials chosen due to their assumed antimicrobial properties [16,17], brass and copper, had consistently higher concentrations of *Legionella* in biofilms than on the cast iron coupon. While this may have been due to a combination of replication, different rates of colonization/release, or impacts on culturability within biofilms formed on cast iron, it appears that *Legionella* may have increased affinity for these presumed antibacterial materials than previously thought. Overall, *Legionella* concentrations were highest on the PVC coupon, although the final recorded concentrations were similar for PVC, brass, and copper. These results suggest that, while pipe material may be a factor in the long-term survival of *Legionella* populations, the dynamics of *Legionella* persistence in distribution systems may rely more significantly on other factors, such as pipe age, water quality, and microbial community, which were unaccounted for in this batch study. The net concentration changes were similar between water and biofilms for each coupon, with the exception of cast iron, which demonstrated an additional 1-log reduction compared to the other coupons. Overall, steady reductions in *Legionella* concentration were measured for the water surrounding each material, while concentrations in biofilms experienced more drastic fluctuations. This discrepancy between concentrations over time in biofilms and water suggests that dynamics such as biofilm release/re-colonization, replication, or cell cycling (including transitions to viable but non-culturable states) may have been occurring, potentially most noticeably within biofilms on the cast iron coupon. As expected, *Legionella* population dynamics substantially varied amongst biofilms and water, characterized by periods of concentration increase, stability, and sharp decrease in biofilms, as opposed to steady decreases observed in water samples.

### 3.3. Growth and Survival of *Legionella* in a Model Drinking Water Distribution System

The initial fluctuation in the concentration of *Legionella* over the first 10 days after inoculation can be explained by dispersal throughout the MDS and colonization of biofilms. The spikes in concentration occurring at days 58 and 131 were potentially due to release of organisms from biofilms resulting from incidental agitation while removing pipe segments for biofilm sampling. This suggests that analogous disruption events within a drinking water distribution system could cause increases in *Legionella* concentration in flowing water, resulting in an increased risk of exposure to *Legionella*. Because the vast majority of *Legionella* in the MDS were always biofilm associated (the system contained substantially more cm^2^ of surface area than mL of flowing water), the results also suggest that monitoring of only water samples may not provide an accurate estimation of the true level of contamination within a distribution system. Despite the two sampling ports used being located on opposite ends of the MDS with differing flow conditions, similar concentrations (1.44 × 10^3^ CFU/mL and 1.48 × 10^3^ CFU/mL) of *Legionella* were seen in the flowing water samples collected two hours after inoculation, suggesting a rapid and even dispersal throughout the MDS and within dead ends. This was further supported by the fact that mean *Legionella* concentrations in samples collected from these two flow conditions remained relatively similar throughout the study. In addition, similar concentrations were observed in water samples collected before and after flushing the ports. It should be noted that the largest variation in water concentrations was measured in the samples collected after chlorination on day 131, possibly related to both differential dispersal of chlorine and sedimentation depending on flow conditions of pipe sections. After fluctuations, *Legionella* concentrations in both flowing water and biofilms of the MDS reached stable levels for extended periods of time, with an initial increase, then decrease, followed by stabilization. However, in biofilms the periods of increase and decrease occurred 14 days earlier than in the flowing water samples. Chlorination resulted in significant reduction of *Legionella* within biofilms (1.4 log, *p*-value: 0.0419) and flowing water (0.5 log, *p*-value: 0.0314). The difference in the effectiveness of chlorination against *Legionella* present in water and biofilm seems contrary to the notion that biofilms typically provide additional protection from disinfection [18]. The reactive properties of the chlorine itself (alongside the previously mentioned physical disruption) may have played a role in increasing the concentration of *Legionella* in the flowing water in the system by inducing the release of *Legionella* containing biofilm fragments into the flowing water. This demonstrates a potential scenario in which the concentration of *Legionella* or other biofilm-dwelling pathogens could increase in the flowing water of a distribution system following increased disinfection. The eventual increase and stabilization observed for concentrations in both biofilms and flowing water following chlorination coincides with previous evidence highlighting the difficulty of treating distribution systems for *Legionella* [19].

### 3.4. Presence of *Legionella* in Water Meter Biofilms

The fact that biofilms collected from the water meters tested positive for the presence of *Legionella* demonstrates a practical approach for future studies on the occurrence and prevalence of this pathogen in drinking water distribution system biofilms (Table 1). While not perfectly representative of biofilms found elsewhere in a distribution network, those within the water meters present a source of samples widely accessible and numerous in any major metropolitan area. The possible effects of brass (as opposed to other distribution system pipe materials) on the survival and biofilm colonization of *Legionella* could have impacted the results of this study. These effects may be negligible, as previous experiments in this study demonstrated the capability for *Legionella* to associate within biofilms formed on brass in a manner similar to other common pipe materials (Figure 2). More than half of the water meter biofilms positive for *Legionella* also tested positive for *L. pneumophila*, indicating that the species of *Legionella* most commonly implicated in human disease may also be relatively common in relation to other members of the genus in drinking water distribution systems. In addition, the high occurrence in drinking water distribution systems across the United States [12] suggests that a widespread *Legionella* contamination may be typical. A number of explanations could be considered as to why Area B contained no water meters positive for *Legionella*, whereas those from Area A tested positive for both *Legionella* and *L. pneumophila*. While both Area A and Area B receive their water from the same three sources in central Arizona, the proportions from each source are not identical and varied during different seasons. In addition, Area B consists of a newer pipe network, and water treatment methods differed between the two. Further examination of these and other factors could determine how drinking distribution systems may create environments that either inhibit or facilitate *Legionella* growth and survival.

## 4. Materials and Methods 

### 4.1. Media and Laboratory Strain of *Legionella*

All *Legionella* cultures were prepared according to the methods previously described by the United States Centers for Disease Control and Prevention [20]. Culturing was performed using buffered charcoal yeast extract agar medium (BCYE) (BCYE Agar Base, Benson, Dickson, and Company, Franklin Lakes, NJ, USA). BCYE was prepared with the following supplements: 0.3% glycine, 100 units/mL polymixin B, 5 µg/mL vancomycin, and 80 µg/mL cyclohexamide, and 0.4g l-cysteine HCl. *L. pnuemophila* stock cultures were grown at 37 °C with agitation over 72 h in Buffered Yeast Extract (BYE) liquid medium. Cell concentration in stock cultures was estimated using optical density measurement at 600 nm. The spread plate technique was used to quantify *Legionella* after incubation at 37 °C for 72 h, with up to seven additional days as needed. All laboratory experiments utilized the *L. pneumophila* serogroup 1 strain Knoxville-1 (American Type Culture Collection 33153).

### 4.2. Molecular Detection of *Legionella*

DNA extraction was performed on drinking water meter biofilm samples using a ZYMO Research yeast/bacterial DNA extraction kit (Zymo Research Corporation, Irvine, CA, USA). *Legionella* spp. and *L. pneumophila* specific primers were used in this study (Table 2). For both primer sets, PCR amplification mixtures consisted of: 12.5 µL Promega GoTaq Green MasterMix (Promega Biosciences LLC., San Luis Obispo, CA, USA), 10 µL DNA template, and 0.13 µM each primer, with a final reaction volume of 25 µL. Gel electrophoresis was performed in a 40 mL 1% agarose gel containing 2 µL of 10,000X Invitrogen SYBR Safe DNA Gel Stain (Life Technologies Corporation, Carlsbad, CA, USA) to detect PCR products. Sequencing was performed on PCR products to confirm the presence of *Legionella* or *L. pneumophila*.

**Table 2:** PCR primers used in the study.

Primers	Sequences (5′→3′)	Gene Amplified	Amplicon Length	Reference
LEG-226	AAGATTAGCCTGCGTCCGAT	*Legionella* 16S rRNA	654 bp	[21]
LEG-858	GTCAACTTATCGCGTTTGCT			
LpneuF	CCGATGCCACATCATTAGC	*L. pneumophila mip*	150 bp	[21]
LpneuR	CCAATTGAGCGCCACTCATAG			

### 4.3. Survival of *Legionella* in Tap Water at 4 °C, 25 °C, and 32 °C

A series of 50-mL polystyrene conical tubes were filled with 50 mL of dechlorinated tap water from the city of Tempe, AZ, USA. Following inoculation with 3 × 10^7^ *L. pneumophila* cells, duplicate tubes were incubated at 4 °C, 25 °C, and 32 °C. The temperatures chosen for this experiment were intended to represent the range of temperatures commonly recorded in water distribution systems across the United States. The study parameters simulate two temperature conditions possible in an actual distribution system where growth of *Legionella* may occur (25 °C and 32 °C) and a condition under which replication is impossible (4 °C) [13]. Over the course of 97 days, the tubes were sampled periodically following mixing and colony forming units (CFU)/mL were determined via the spread plate technique on BCYE agar media.

### 4.4. Association of *Legionella* within Biofilms on a Variety of Pipe Coupons

A series of 5-L polyethylene containers were filled with 1 L of tap water from the city of Tempe, AZ, USA. Single coupons of PVC, cast iron, copper, and brass were placed in the containers and incubated at 25 °C for three weeks to allow biofilm development. The materials studied were chosen to represent a range of pipes and water fixtures commonly found in drinking water distribution systems in the United States. Following the initial biofilm incubation, 3 × 10^8^ *L. pneumophila* cells were spiked in each container. One-milliliter water samples and 2 cm^2^ biofilm samples were collected from different sections on the coupons in each container periodically over 94 days. Prior to sampling, the water in each container was gently agitated to mix in the suspended *Legionella* while maintaining biofilm integrity. Biofilm samples were collected with sterile cotton swabs and resuspended in sterilized deionized water. CFU/mL and CFU/cm^2^ were determined for each sample via spread plate technique on BCYE agar medium.

### 4.5. Growth and Survival of *Legionella* in a Model Drinking Water Distribution System 

The ability of introduced *Legionella* to colonize and survive within distribution system water and biofilms was established using a laboratory scale model water distribution system. The MDS has a loop with PVC piping, consisting of a 5.5 m long and 5.1 cm diameter main pipe, with a 1.4 m long dead end, and a total volume of 50 liters. Distribution mains and dead-end lines consisted of six and two removable pipe sections (65 cm each in length), respectively. The main pipe is connected to a reservoir. A self-priming, thermally protected, magnetic-drive pump (Little Giant Pump Company, Oklahoma City, OK, USA) continuously re-circulated water between the main loop and the reservoir. Pressure, flow rate (0.304 m/s), and temperature (25 °C) were kept constant through external controls. The MDS had been used continuously for 11 years and had well established biofilm communities before the start of this experiment.

City of Tempe, AZ, USA tap water was circulated in the MDS, following dechlorination. PVC coupons 2.5 cm in diameter and 5 cm in length were suspended in the reservoir for several weeks to allow biofilm formation. Prior to inoculation with a laboratory strain of *L. pneumophila*, water and biofilm samples were tested to confirm the absence of culturable *Legionella* in the MDS. After inoculation with approximately 10^7^ *L. pneumophila* cells, water and biofilm samples from the MDS were periodically collected over 131 days and cultured on BCYE to determine *Legionella* concentrations. Water samples were collected from two sampling ports: (1) one located in a dead end segment of the system approximately 3 m from the reservoir and (2) one located in an open segment of the system approximately 1.2 m from the reservoir. Two 5-mL water samples were collected from each port for each sampling event, one before and one after flushing 1 L of water, with *Legionella* concentrations averaged between the two. This form of sampling was performed to assess any variation between *Legionella* in the samples due to stagnant water or biofilms in the ports. When necessary, water samples were concentrated via the membrane filtration technique using 0.45-micron pore size filters resuspended in 10 mL of sterilized deionized water. Biofilm samples were collected from PVC coupons using sterile cotton swabs and resuspended in sterilized deionized water prior to spread plating. After 131 days, the MDS was treated with 5 mg/L of chlorine introduced into the reservoir. Two hours later, water samples were collected along with biofilm samples from a coupon, a dead-end section, and an open end section of pipe.

### 4.6. Presence of *Legionella* in Residential Water Meter Biofilms

Residential drinking water meters were collected from two general areas located in central Arizona: 35 from area A and 32 from area B. Sampling occurred over a period of approximately one month during June/July 2011. Collection was possible due to water meter recycling programs in which water utilities replace mechanically worn meters with new ones. All water meters collected were made of brass, with some containing rubber washers on their inlet rims. Upon removal by utility personnel, meters were submerged in tap water and transferred to the environmental microbiology laboratory at Arizona State University. Biofilm samples were collected from the inlet of each meter within two hours of removal. Approximately 40 cm^2^ of biofilm were collected using sterilized nylon wire brushes and resuspended in 20 mL of sterile water. DNA extraction was performed on 750 µL of this suspension, followed by PCR amplification using specific primers for *Legionella* spp. and *L. pneumophila.* PCR reactions producing bands visible on a DNA gel were sequenced to identify the presence of *Legionella* or *L. pneumophila*. Biofilm samples positive for *Legionella* were cultured on BCYE via the spread plate technique.

### 4.7. Data Analysis

Excel (Microsoft Corporation, Redmond, WA, USA) was used for all data analysis and graph generation. Student’s *t*-tests were performed to determine the statistical significance of select data.

## 5. Conclusions

With legionellosis cases continually on the rise in the United States and across the world, the development of appropriate public health protocols to curtail the incidence of this theoretically preventable disease is more important now than ever. Despite the fact that a substantial proportion of efforts focus on monitoring and treatment procedures aimed at preventing *Legionella* contamination of in-premise plumbing, the dispersal and contamination of *Legionella* in these systems is clearly an important factor to consider. The very water distribution systems that introduce these pathogens to building plumbing systems are a logical target to help prevent legionellosis incidence. By examining *Legionella* survival in tap water, association in pipe material biofilms, interactions in a model distribution system, and prevalence in drinking water distribution system biofilms, the results of this study provide valuable information relevant to the design of monitoring and control procedures for *Legionella* in public drinking water.
